# The development of cervical intraepithelial neoplasia in three members of the same family: a case report

**DOI:** 10.3389/frph.2025.1542480

**Published:** 2025-05-13

**Authors:** M. A. Vinokurov, A. V. Minaeva, G. V. Leshkina, T. N. Romanyuk, K. O. Mironov, V. G. Akimkin

**Affiliations:** ^1^ Central Research Institute for Epidemiology, Federal Service for Surveillance on Consumer Rights Protection and Human Wellbeing, Moscow, Russia; ^2^ Medical Institute, Peoples' Friendship University of Russia Named After Patrice Lumumba, Moscow, Russia

**Keywords:** cervical intraepithelial neoplasia (CIN), high grade squamous intraepithelial lesion (HSIL), cervical cancer, HPV—human papillomavirus, family case report, genetic predisposition, single nucleotide (NT) polymorphism (SNP)

## Abstract

**Background:**

Cervical cancer is the fourth most common cancer and cause of cancer-related death among women globally. Familial cases of cervical cancer highlight the potential role of genetic factors in its development. This study aims to present a clinical case of cervical intraepithelial neoplasia (CIN) affecting a woman and her two daughters.

**Case description:**

This report describes a familial case involving three patients diagnosed with cervical lesions: (1) Patient A: A 27-year-old woman presented with complaints of postcoital bleeding. She was diagnosed with high-grade squamous intraepithelial lesion (HSIL, CIN3). Radiowave conization of the cervix was performed, and histological examination confirmed the diagnosis of CIN3. (2) Patient B: A 25-year-old woman, the sister of Patient A, also presented with contact bleeding. She was similarly diagnosed with HSIL (CIN3). A radiowave conization procedure was performed successfully, with histopathological analysis confirming the diagnosis. (3) Patient C: A 52-year-old woman, the mother of Patients A and B, was diagnosed with low-grade squamous intraepithelial lesion (LSIL, CIN1) following cytological examination. She declined further diagnostic and therapeutic interventions. Genetic testing for all three patients revealed the presence of risk alleles associated with cervical cancer predisposition (rs10175462, rs1048943, rs4646903) and the absence of protective genotypes.

**Discussion:**

Familial cases of CIN are rare and suggest a potential genetic predisposition to the disease. The identification of common genetic polymorphisms underscores the role of hereditary factors in cervical cancer pathogenesis. These findings emphasize the importance of incorporating family history and genetic assessments into screening, diagnosis, and treatment strategies.

**Conclusion:**

This case highlights the significant influence of genetic factors in the development of cervical intraepithelial neoplasia. It underscores the need for further research to enhance strategies for early detection, prevention, and management of cervical cancer in individuals with elevated genetic risk.

## Background

Cervical cancer ranks as the fourth most common and deadly cancer among women worldwide. In 2020, approximately 604,000 new cases were diagnosed, and more than 340,000 women died from the disease, accounting for 7.7% of all cancer-related deaths globally (1). Between 2009 and 2019, the incidence of cervical cancer in Russia increased by nearly 12%, highlighting the growing significance of this health concern. The highest incidence rates are observed among women aged 30–44 years (2).

The primary etiological factor in cervical cancer is infection with high-risk types of human papillomavirus (HPV) (3). However, genetic predisposition also contributes as a significant risk factor. Familial occurrences of cervical cancer suggest an important genetic component in its development. Analysis of the Swedish Family-Cancer Database indicates that 64% of cervical cancer risk stems from hereditary factors, while 36% is associated with environmental influences (4). This underscores the critical role of inherited factors in the pathogenesis of cervical cancer.

This study aimed to examine a clinical case of cervical intraepithelial neoplasia (CIN) affecting a woman and her two daughters.

## Materials and methods

Cytological and histopathological examinations were conducted in various large independent laboratories accredited by the Russian quality assurance system, as well as by certified external quality control centres in Russia. Standard Papanicolaou (PAP) staining and hematoxylin and eosin (H&E) staining techniques were used. Prepared slides were independently reviewed by two pathologists, who were unaware of the identity of the patient. DNA extraction from blood samples and cervical specimens, collected in BD SurePath transport medium (BD Diagnostics), was conducted using RIBO-Prep kits (registration certificate FSR 2008/03147) and AmpliSens® DNA-sorb-D kits (registration certificate RZN 2015/3503). High-risk HPV types were assessed using AmpliSens® HR HPV screen-titer-14-FL kits (registration certificate RZN 2017/5387) and HR HPV genotype titer FL kits (registration certificate RZN 2017/6533), enabling genotyping of 14 high-risk HPV types (16, 18, 31, 33, 35, 39, 45, 51, 52, 56, 58, 59, 66, and 68), alongside the Digene Hybrid Capture® 2 assay. Genetic analysis involved polymerase chain reaction (PCR) to genotype nine single nucleotide polymorphisms (SNPs) using TaqMan technology (Thermo Fisher Scientific) on DNA extracted from peripheral blood samples. These genetic markers were selected based on prior studies (5). All procedures adhered to clinical guidelines and were conducted in a licensed laboratory.

## Case description

**Figure 1:**
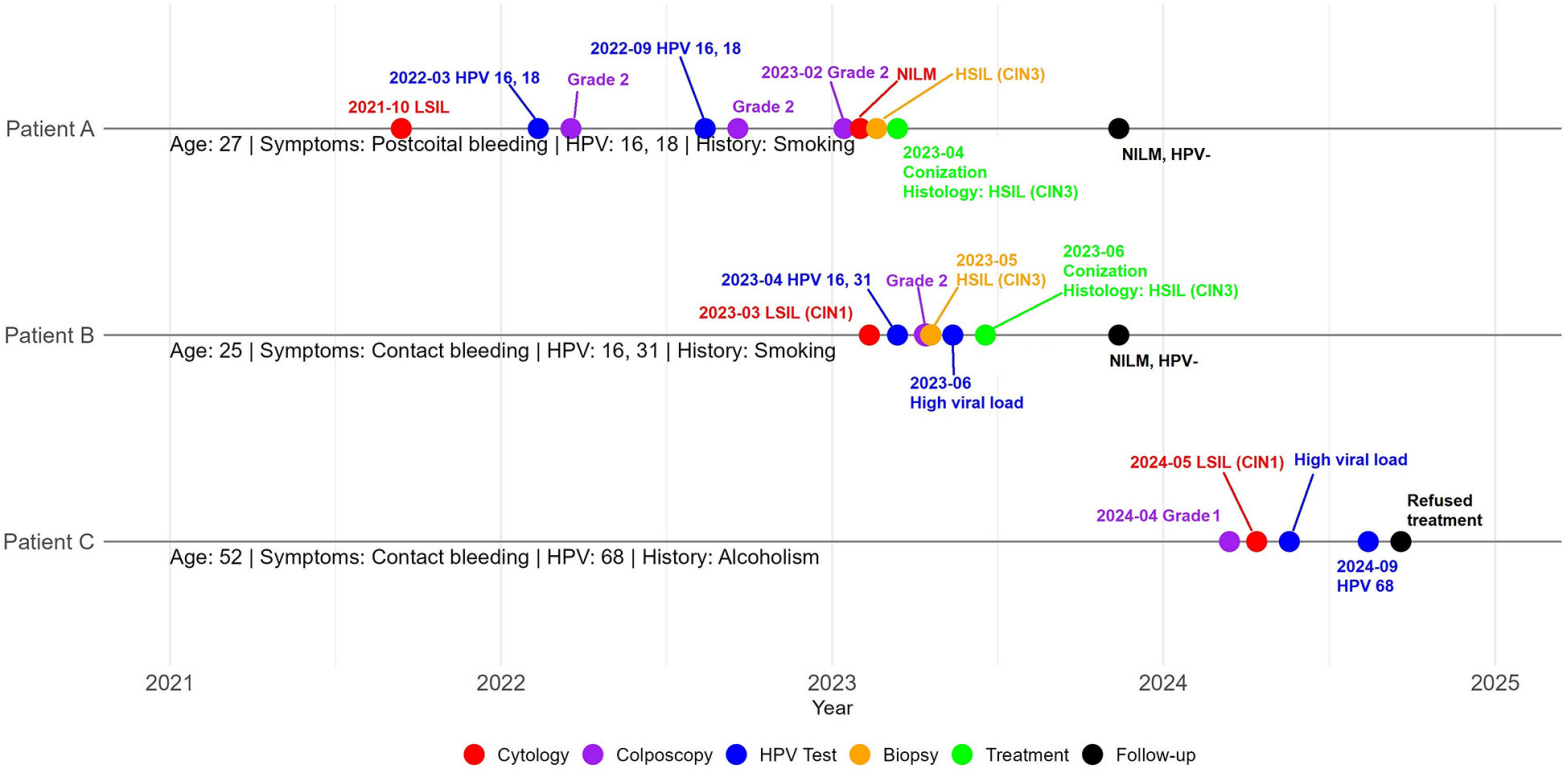
Clinical management of patient A, her sister patient B, and their mother patient C.

We present a familial case of cervical lesions affecting three patients: Patient A, her sister Patient B, and their mother Patient C (Figure 1).

**Patient A**, a 27-year-old woman (gravida 1, para 1), presented in March 2023 with abnormal post-coital cervical bleeding.

She began smoking at the age of 19 and has been smoking one pack of cigarettes daily for the past eight years. Her medical history includes multiple gynecological conditions, such as cervical ectopy, bacterial vaginosis, candidiasis, and a functional ovarian cyst. In 2015, she was diagnosed with chlamydia. Additionally, she suffers from chronic gastritis (Helicobacter pylori positive), chronic hemorrhoids, and clinical depression, for which she has been receiving antidepressant treatment for four years.

Patient A experienced menarche at age 12, with menstrual cycles lasting 5–6 days every 31–35 days. She became sexually active at the age of 16 and was in a monogamous relationship for three years prior to presentation. During her pregnancy, she gained 29 kilograms and delivered at 42 weeks' gestation via induced labor. The male neonate had Apgar scores of 4 at 1 min and 5 at 5 min, requiring palliative care due to birth trauma.

Anthropometric data: Height 164 cm, weight 75.5 kg, body mass index (BMI) 28.07 kg/m^2^. Examination of the mammary glands and lymph nodes was unremarkable. Pelvic examination revealed a normal-sized uterus with no tenderness on palpation. The cervix appeared erythematous with visible contact bleeding, consistent with the patient's chief complaint.

In 2020, PCR testing for 14 different HPV types yielded negative results. However, in October 2021, a cytological examination of a cervical smear revealed a low-grade squamous intraepithelial lesion (LSIL), and the patient was advised to undergo regular monitoring.

By March 2022, PCR analysis detected high-risk HPV types 16 and 18 at concentrations of 8.1 and 5.1 log copies of the viral genome per cell, respectively. Conventional oncocytological examination revealed exo-/endocervicitis, while colposcopic examination showed abnormal findings classified as Grade 2 and LSIL.

A follow-up examination in September 2022 revealed a decrease in the concentration of HPV type 16–5.6 log copies, while the level of HPV type 18 remained stable.

In February 2023, colposcopy confirmed abnormal findings classified as Grade 2 (coarse mosaic) in transformation zone 1 (TZ1). Despite these findings, cytological examination showed no intraepithelial lesion or malignancy (NILM), and a transvaginal ultrasound revealed no abnormalities.

According to the American Society for Colposcopy and Cervical Pathology (ASCCP) guidelines, the immediate risk of CIN3 or higher was estimated at 15.6%, with a five-year risk of 24.1%.

**Figure 2:**
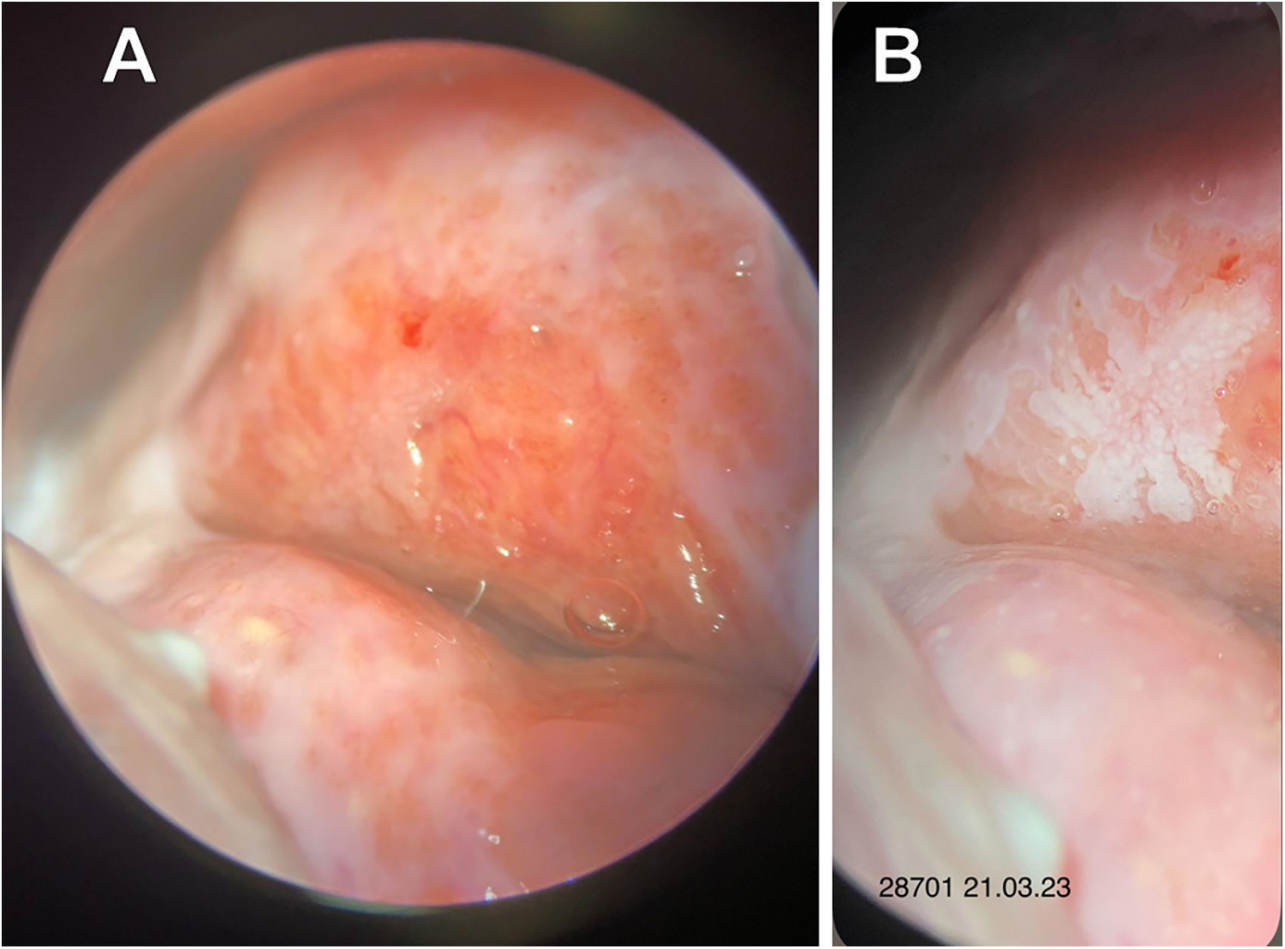
Extended colposcopy of patient A. **(A)** Native view **(B)** after acetic acid application: abnormal colposcopic findings grade 2 (Coarse Mosaic).

On March 21, 2023, a multifocal targeted biopsy was performed under colposcopic guidance (Figure 2).

Histopathological examination revealed ectopia of the endocervical epithelium associated with a HSIL (CIN3).

On April 14, 2023, the patient was assessed by an oncology specialist and subsequently underwent radiofrequency conization of the cervix along with curettage of the cervical canal. Histological analysis confirmed CIN3, with clear resection margins devoid of neoplastic involvement.

Follow-up assessments conducted at 3, 6, and 12 months post-conization included PCR testing for 14 high-risk HPV genotypes, Hybrid Capture 2 (HC2) assay, and PAP-test. All results were negative for cytological abnormalities and HPV infection.

**Patient B**, a 25-year-old woman (gravida 0, para 0), and the sister of Patient A, presented in March 2023 with complaints of contact bleeding.

Menarche occurred at age 13, with menstrual cycles lasting 3–4 days every 28–90 days, indicative of menstrual irregularity. She experienced coitarche at age 17 and has had three sexual partners. The patient reports using a glycerin-based vaporizer containing 2% nicotine and consuming two bottles of alcoholic beverages weekly. Her medical history includes cervical ectopy, urinary tract calculi, pityriasis versicolor, and common childhood illnesses such as rubella, measles, varicella, and mumps. She has been undergoing antidepressant treatment for clinical depression for the past two years.

Physical examination revealed a height of 165 cm, weight of 52 kg, and a body mass index (BMI) of 19.01 kg/m^2^. Examination of the mammary glands and lymph nodes was unremarkable. Bimanual pelvic examination showed a non-tender uterus and adnexa, with no palpable masses. The cervix exhibited mild friability and hyperemia, correlating with reported contact bleeding.

On March 12, 2023, a cytological examination identified a LSIL (CIN1). Subsequent PCR analysis on March 18 detected HPV type 16 at 6.7 log copies per DNA sample and HPV type 31 at 4.9 log copies per DNA sample. A colposcopic examination on April 1 revealed abnormal findings classified as Grade 2 (coarse acetowhite epithelium). According to the ASCCP guidelines, the immediate risk of CIN3 or higher was estimated at 11%.

**Figure 3:**
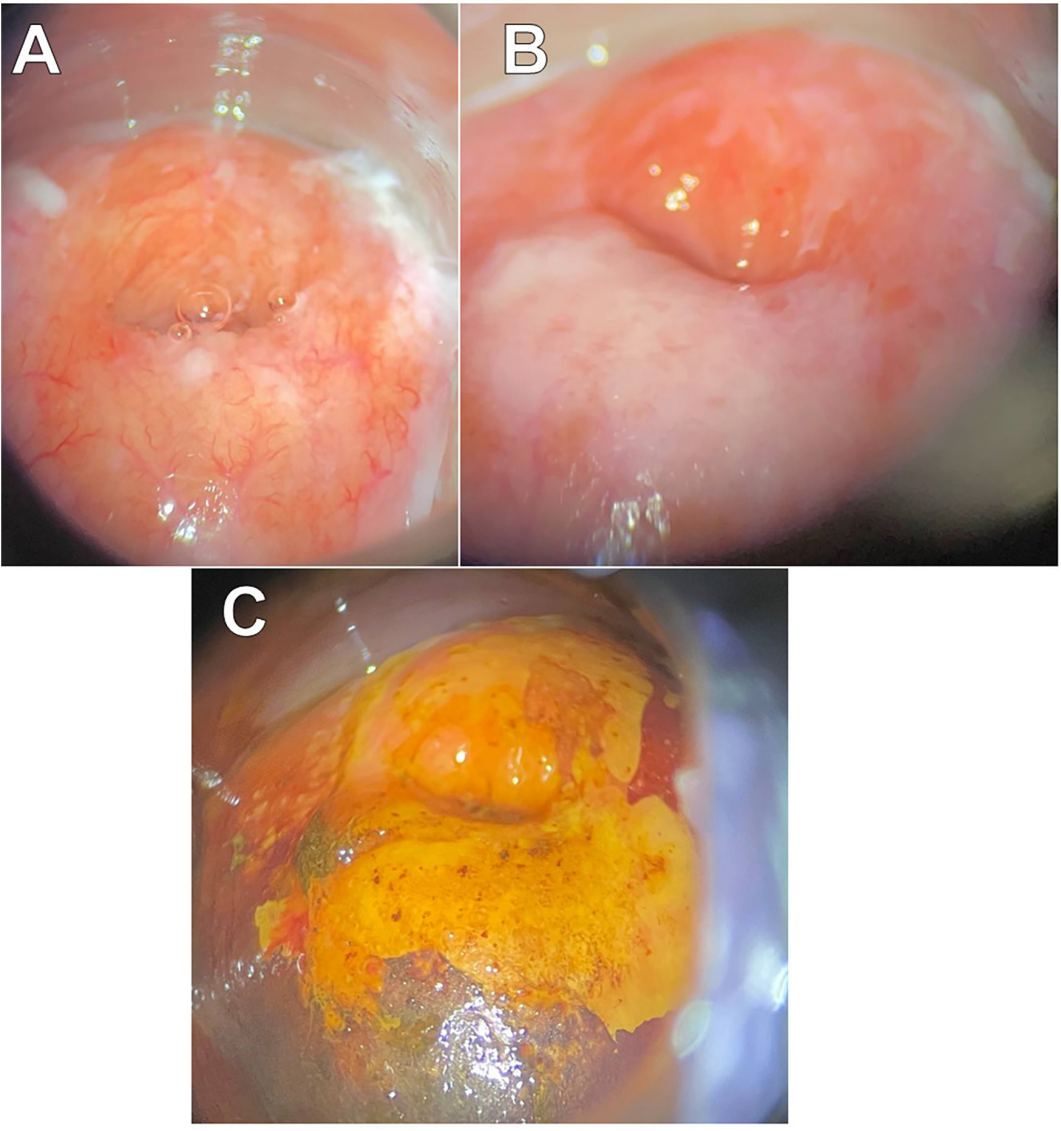
Extended colposcopy of patient B. **(A)** Native view **(B)** after acetic acid application: abnormal colposcopic findings grade 2 (coarse acetowhite) **(C)** Schiller's Test.

On May 23, 2023, a multifocal biopsy was performed (Figure 3), and histological analysis confirmed the presence of a HSIL (CIN3).

On June 4, 2023, the HC2 test revealed a significant viral load of 536.36 relative units. Following this, on June 19, the patient underwent radiofrequency conization of the cervix and curettage of the cervical canal. Histological examination confirmed pronounced dysplastic changes consistent with CIN3, with resection margins free from neoplastic involvement.

A three-month follow-up PAP-test on August 14, 2023, revealed NILM, indicating a successful treatment outcome.

Based on the findings in Patients A and B, their mother, **Patient C**, a 52-year-old woman (gravida 4, para 2), was evaluated in April 2024 due to complaints of contact bleeding.

Menarche occurred at age 14, with regular menstrual cycles lasting 3–4 days every 28–30 days. She became sexually active at age 17 and has had six lifetime sexual partners, all in monogamous relationships. Her medical history is notable for chronic alcoholism and chronic pancreatitis. She also experienced varicella (chickenpox) during childhood.

Anthropometric measurements indicated a height of 162 cm, weight of 61 kg, and a BMI of 23.2 kg/m^2^. Examination of the mammary glands and regional lymph nodes was unremarkable. Bimanual examination indicated a normal-sized uterus and no adnexal abnormalities. The vaginal mucosa appeared atrophic, consistent with postmenopausal status, with no overt lesions beyond colposcopic findings.

**Figure 4:**
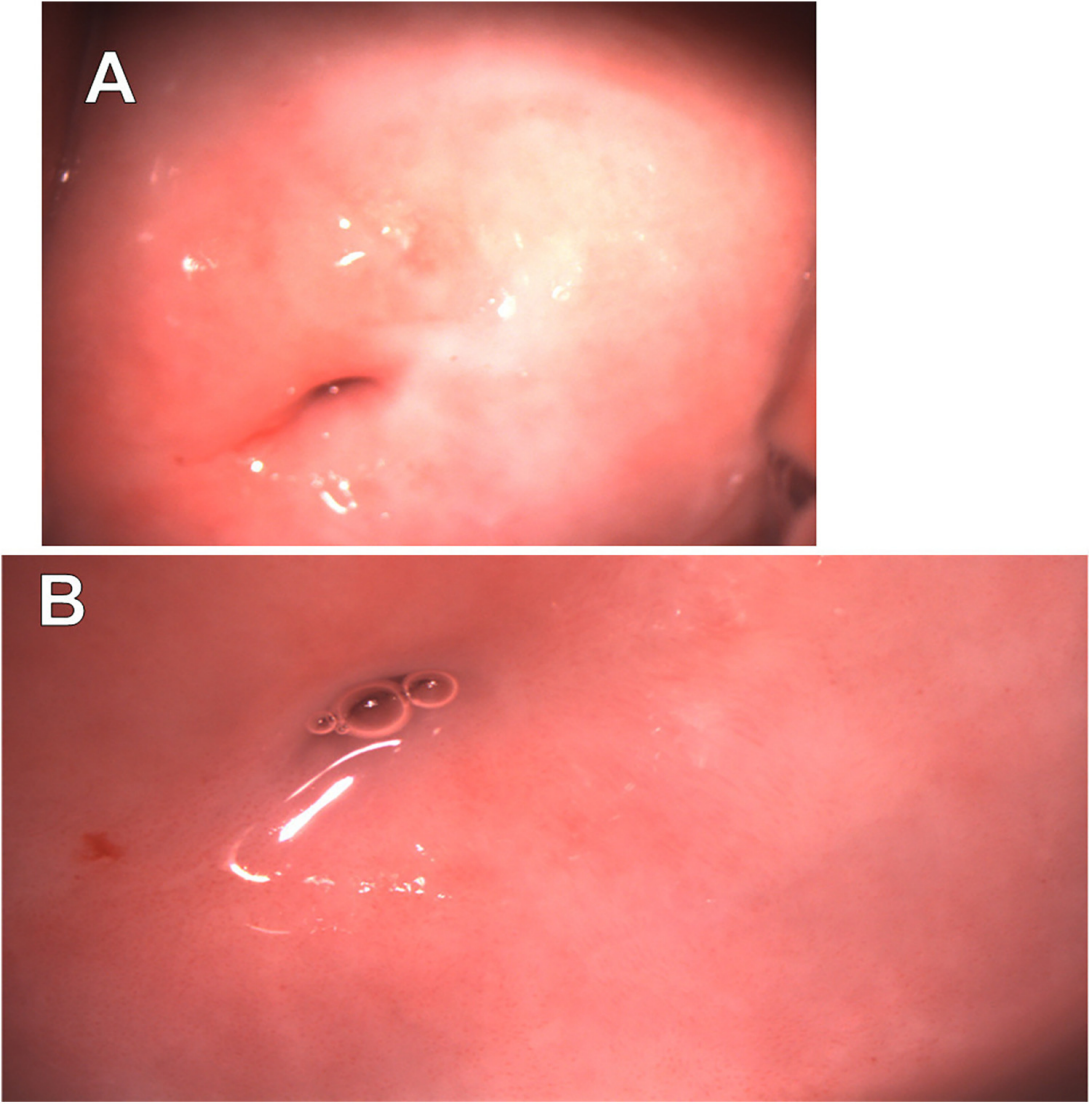
Extended colposcopy of patient C. **(A)** Native view **(B)** after acetic acid application: abnormal colposcopic findings grade 1 (fine punctuation), TZ3.

On April 19, 2024, colposcopic evaluation revealed non-specific findings, including abnormal colposcopic features classified as Grade 1 (fine punctation) and a TZ3 (Figure 4).

On May 27, 2024, cytological examination revealed the presence of a LSIL (CIN I). On the same day, the HC2 assay detected a high-risk HPV strain with a viral load of 77.12 relative units. Subsequently, a PCR analysis performed on September 15, 2024, confirmed the presence of HPV type 68, with a viral load of 5.5 log copies per DNA sample.

Due to the patient's refusal to pursue further treatment and evaluation, available clinical management options are currently limited.

A genetic risk assessment was conducted utilizing PCR-based techniques to identify alleles associated with cervical cancer susceptibility at specific loci: *rs1048943*, *rs55986091*, *rs138446575*, *rs2516448*, *rs9271898*, *rs73728618*, *rs10175462*, *rs1801133*, and *rs4646903* (5).

• Patient A was identified as carrying risk alleles at loci *rs10175462* and *rs1048943*. Additionally, she lacked protective genotypes at variants *rs2516448*, *rs9271898*, and *rs55986091*, which are associated with a reduced risk of cervical cancer.
• Patient B carried risk alleles at loci *rs10175462*, *rs4646903*, and *rs1048943*, mirroring the genotype of Patient A, with the absence of protective variants.
• Patient C exhibited an identical genotype to Patient B, sharing the same risk alleles and the absence of protective genotypes.

Collectively, these genetic findings suggest that all three patients possess a significantly elevated genetic risk for cervical cancer.

## Discussion

We present a familial case involving cervical lesions in three patients: Patient A, diagnosed with HSIL (CIN3); her sister, Patient B, also diagnosed with HSIL (CIN3); and their mother, Patient C, who was suspected to have a cervical lesion but declined further examination and treatment.

HPV is highly prevalent in the general population, with approximately 80% of sexually active individuals becoming infected with at least one high-risk HPV type during their lifetime (6). However, not all individuals infected with HPV develop cervical cancer. Approximately 2%–3% of HPV-infected individuals develop LSIL (7), and progression to more severe lesions, such as HSIL, is observed in approximately 8.7% of LSIL cases (8). In the absence of intervention, invasive cervical cancer develops in approximately 20% of HSIL cases within 15–20 years (9). Therefore, the estimated risk of developing cervical cancer among HPV-infected individuals ranges from 0.028% to 0.042%. Based on these estimates, the probability of observing two cases of HSIL and one case of cytological LSIL occurring simultaneously within the same family is exceedingly low, with a combined probability of approximately 0.000027%. Despite this low probability, a review of the literature consistently identifies cases of cervical lesions occurring within families.

Hereditary susceptibility and a genetic contribution to cervical cancer development were first identified in 1959, in a documented case involving three sisters with cervical abnormalities. The first sister was diagnosed with cervical erosion and precancerous lesions, the second with invasive cervical carcinoma, and the third with cancer exhibiting deep stromal invasion (10).

A study on familial cervical cancer in three Dutch families investigated the potential genetic predisposition to the disease. In one family, a 24-year-old patient diagnosed with cervical cancer had both a grandmother and an aunt diagnosed with the same condition, suggesting a possible genetic linkage (11).

Several studies have explored the familial occurrence of cervical cancer, particularly in families where multiple members, such as three sisters, have developed the disease. These studies have attempted to link the familial occurrence of cervical cancer to genes in the human leukocyte antigen (HLA) system. Despite the absence of traditional epidemiological risk factors, a genetic contribution is suspected. Although no firm association with specific HLA patterns has been established, certain *HLA* genotypes may influence susceptibility to oncogenic viruses. Ongoing investigations into the genetic predisposition of these families, as well as further surveillance of younger siblings, are warranted (12).

Data from the German Cancer Registry indicate that approximately 22% of patients with cervical cancer have a family history of the disease. Among these individuals, 50% have a mother who also had cervical cancer, and 11% have more than one immediate family member affected (13).

A study conducted by the Swedish Cancer Registry identified a pattern of cancer incidence within families, showing that women from certain families have a relative risk of cervical cancer 1.5–2.3 times higher than the general population. This increased risk is comparable to that associated with hereditary breast cancer, which has an established genetic component. The evidence from familial cervical cancer cases suggests a significant genetic influence on the development of this condition (14).

Cervical cancer development is closely associated with various factors, including lifestyle choices and deficiencies in healthcare systems. Known risk factors include prolonged use of oral contraceptives, smoking, lack of vaccination, and infections with *Trichomonas vaginalis* and *Chlamydia trachomatis* among others (15). Infection with high-risk HPV types (e.g., 16, 18, 31, 33, 35, 39, 45, 51, 52, 56, 58, 59, 68, 73, and 82) (16) significantly contributes to the development of the disease. Furthermore, the familial occurrence of cervical lesions suggests that hereditary factors play a substantial role in its pathogenesis.

Research into the genetic factors of cervical cancer has identified potential susceptibility variants in tumor suppressor genes and regulatory genes involved in cell cycle control and DNA repair (17).

In our study, patients were tested for single nucleotide polymorphisms at the following loci: *rs1048943*, *rs55986091*, *rs138446575*, *rs2516448*, *rs9271898*, *rs73728618*, *rs10175462*, *rs1801133*, and *rs4646903*. According to meta-analyses and genome-wide association studies, these loci are associated with cervical cancer development. Some patients were found to carry genotypes linked to an increased risk of cervical cancer. Several of these polymorphisms are located in the *CYP1A1* gene, which participates in the metabolism of xenobiotics, including carcinogens from tobacco smoke. Considering the smoking habits of the patients, this may represent an additional risk factor. Other polymorphic variants are located in genes involved in immune regulation and inflammation, particularly within the HLA family (5) suggesting that variations in these genes may affect the immune response to HPV infection.

In addition to genetic predisposition, environmental and behavioral factors likely contributed to the development of CIN in this family. Patients A and B were active smokers, a known risk factor for cervical cancer that may enhance the carcinogenic effects of HPV through CYP1A1-mediated metabolism of tobacco-derived xenobiotics (18, 19). Despite the late detection of LSIL, Patient C, along with a family history, had other risk factors that could increase the chance of developing cervical pathology. These included chronic alcoholism and having multiple sexual partners (20, 21). These shared lifestyle factors among family members highlight the interplay between genetics and environment, suggesting that hereditary risk may be amplified by modifiable behaviors (17).

We acknowledge the limitations of our study, including the constraints imposed by the study design and the fact that only a single familial case is described. Future research will aim to expand the sample of familial cases and conduct large-scale epidemiological analyses. Furthermore, the mother's refusal to undergo further examination represents a limitation that may affect the generalizability of our findings. The possibility of a false-positive PAP-test result and the absence of histological confirmation may also impact the validity of our conclusions. The findings of this study highlight the importance of obtaining comprehensive family histories in the management of patients with cervical lesions. Such histories can facilitate the identification of individuals at increased genetic risk and allow for the tailoring of screening programs for these individuals. The results also underscore the need for genetic risk assessment in clinical practice. However, further research is required to integrate genetic assessment methods into routine clinical practice, particularly to identify families with a high genetic predisposition to cervical cancer.

### Patient's perspective

The information provided about hereditary cervical lesions is crucial for understanding the development of the disease and motivating individuals to participate in screening activities. Patient A expressed relief at the successful treatment outcome and emphasized the importance of regular screening, motivated by her family history. Patient B noted that understanding the genetic link encouraged her to pursue treatment promptly, despite initial anxiety. Patient C, while declining further intervention, acknowledged the value of the information for her daughters' health management.

## Data Availability

The original contributions presented in the study are included in the article/Supplementary Material, further inquiries can be directed to the corresponding author.
